# Role of IL-5 in eosinophil-associated diseases and prospects for multi-target therapy

**DOI:** 10.3389/fimmu.2026.1817893

**Published:** 2026-04-10

**Authors:** Weiying Ou, Yaoyao Wang, Chengyu Hou, Shujuan Hou, Yumei Zhou, Ji Wang, Qi Wang

**Affiliations:** 1 National Institute of Traditional Chinese Medicine (TCM) Constitution and Preventive Treatment of Disease, Wangqi Academy of Beijing University of Chinese Medicine, Beijing University of Chinese Medicine, Beijing, China; 2 College of Traditional Chinese Medicine, Beijing University of Chinese Medicine, Beijing, China; 3 College of Traditional Chinese Medicine, Hubei University of Traditional Chinese Medicine, Wuhan, China

**Keywords:** biologics, eosinophil-associated diseases, eosinophils, interleukin-5, multi-target therapy

## Abstract

Eosinophil-associated diseases are a group of inflammatory disorders characterized by abnormal eosinophil infiltration, which significantly impacts patients’ quality of life. Interleukin-5 (IL-5), a critical cytokine that regulates eosinophil development, activation, chemotaxis, and survival, plays a central role in the pathogenesis of these diseases. This review systematically examines the molecular structure and signaling pathways of IL-5, its mechanisms of action in asthma and chronic obstructive pulmonary disease (COPD), and the development and clinical applications of monoclonal antibodies (e.g., mepolizumab, benralizumab, reslizumab) and other biologics targeting IL-5. Although IL-5-targeted therapies have yielded significant results, single-target interventions still exhibit limitations, including insufficient responses in certain patients. To address this, we explore the strategy of multi-target combination therapies, such as the synergistic inhibition of IL-5 with the IL-4/IL-13 and IL-33/ST2 pathways. We also discuss the potential of novel therapeutic approaches, including bispecific antibodies and small-molecule inhibitors. Ultimately, multi-targeted precision therapies, tailored to individual inflammatory phenotypes, are anticipated to represent a new frontier in the management of eosinophil-associated diseases.

## Graphical Abstract

**Figure:**
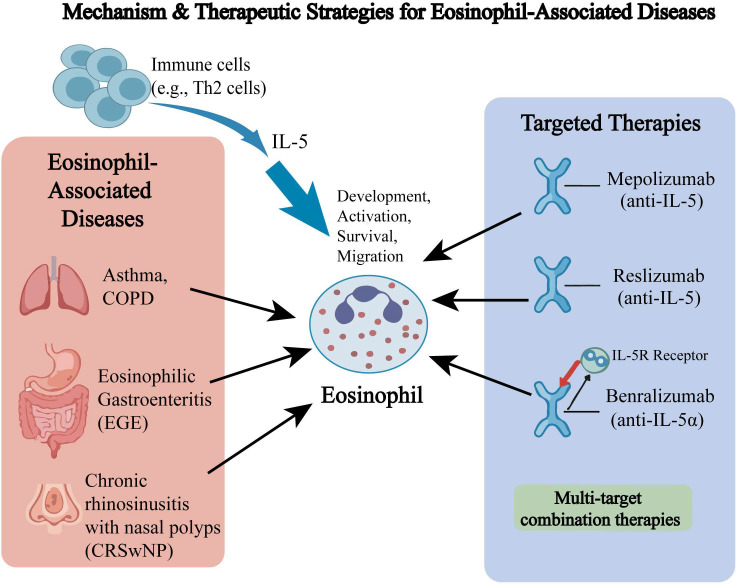

## 1 Introduction

Eosinophil-associated diseases (EADs) are a diverse group of disorders characterized by an abnormal increase in eosinophils and inflammatory infiltrates in tissues or blood (1). These include severe eosinophilic asthma, specific phenotypes of chronic obstructive pulmonary disease (COPD), and eosinophilic gastroenteritis (EGE). EADs often result in significant tissue damage and organ dysfunction, leading to a reduced quality of life for patients and imposing a substantial socio-economic burden.

Interleukin-5 (IL-5) is recognized as the most critical cytokine regulating eosinophils within the immune network (2). A homodimeric protein primarily produced by type 2 helper T cells (Th2) and type II innate lymphoid cells (ILC2s), IL-5 specifically acts on progenitor cells in the bone marrow to promote the maturation, differentiation, and release of eosinophils into peripheral circulation and affected tissues (3). Additionally, IL-5 significantly extends the lifespan of eosinophils and enhances the release of their pro-inflammatory mediators, such as eosinophil cationic protein (ECP), by activating signaling pathways including JAK-STAT, PI3K/AKT, and MAPK (4). These actions contribute to the exacerbation of chronic inflammation.

Clinically, targeting the IL-5 axis has emerged as a paradigm in precision medicine. Monoclonal antibodies that neutralize IL-5 ligands, such as Mepolizumab and Reslizumab, and Benralizumab, which targets the IL-5 receptor subunit, have shown significant efficacy in reducing acute exacerbations and minimizing steroid dependence (5). However, despite advances in biologic therapies, some patients still exhibit inadequate responses or limited efficacy (6). This suggests that eosinophilic inflammation is not driven by a single factor but rather involves a complex interplay of multiple elements, including IL-4, IL-13, and allergens (e.g., IL-33, TSLP) (7–9).

Given the limitations of single-target approaches, the exploration of multi-target combination therapies has become a cutting-edge focus in current research. This review aims to systematically examine the pathogenic mechanisms of IL-5 in various eosinophil-associated diseases, evaluate the pharmacological benefits and clinical limitations of existing biologics, and highlight the potential of novel multi-target therapeutic strategies, such as bispecific antibodies, small-molecule inhibitors, and combined immunomodulation. By integrating multidimensional interventions, the future holds promise for precision management tailored to individual patient inflammatory phenotypes.

## 2 Biological properties of IL-5

### 2.1 Production and structure of IL-5

IL-5 is a cytokine produced as a homodimer and secreted by various cell types, including Th2 cells, mast cells, ILC2 cells, and eosinophils (10). The cytokine consists of two identical polypeptide chains, each approximately 115 amino acids in length. Its three-dimensional structure features four helices that form a stable dimer through a specific folding pattern. This unique structure allows IL-5 to bind to its receptor, IL-5R, thereby activating downstream signaling pathways (11).

### 2.2 IL-5 receptors and signaling pathways

The IL-5 receptor (IL-5R) is a heterodimeric complex consisting of two subunits: α and β. IL-5 activates downstream signaling pathways by binding to its receptor, which includes the high-affinity IL-5 receptor α chain and a shared common β chain (c chain) (12).

IL-5R is primarily responsible for recognizing and binding IL-5 ligands, with expression predominantly found on the surface of eosinophils, basophils, and certain immune cells (13). Notably, IL-5R expression is significantly higher on eosinophils compared to other myeloid cells, making eosinophils particularly sensitive to IL-5 stimulation (14). Activated eosinophils can rapidly downregulate the expression of membrane-bound IL-5R (mIL-5R), a phenomenon that occurs in parallel with CD62L shedding. This suggests that the loss of mIL-5R may serve as a functional marker of eosinophil activation (15).

In addition to eosinophils, recent studies have identified IL-5R expression in epithelial cells, B cells, T cell subsets, and some immune cells within the tumor microenvironment (15). For instance, in pancreatic cancer models, IL-5/IL-5R signaling has been shown to remodel the tumor immune microenvironment, promoting immunosuppressive cell infiltration and accelerating tumor progression (16). Concurrently, IL-5 signaling exerts a pivotal pathophysiological influence on airway epithelial cells (AECs), acting not only as a maturation factor for eosinophils but also as a direct mediator of airway structural remodeling and functional impairment. By activating the intracellular JAK-STAT pathway, IL-5 induces goblet cell metaplasia and significantly upregulates the expression of the mucin MUC5AC, culminating in mucus hypersecretion and the formation of mucus plugs. Furthermore, IL-5 triggers epithelial-mesenchymal transition (EMT) and compromises apical tight junctions, thereby undermining airway barrier integrity and promoting subepithelial fibrosis and persistent structural reorganization (3). Furthermore, single-cell multi-omics analyses have revealed that IL-5 significantly expands the precursor cell pool by driving the transit amplification phase of the eosinophil lineage, without affecting the terminal maturation process. This underscores the dynamic role of IL-5R in regulating cell fate (16).

Together, these findings suggest that the IL-5 receptor is not merely a signaling portal but also a key regulatory hub that controls the strength and duration of cellular responses.

The common β (c) receptor is a key component of signal transduction and serves as a shared chain in the IL-5, IL-3, and GM-CSF receptor complexes. Although it does not directly bind to IL-5, it plays a crucial role in the downstream signaling of these three cytokines (12). This shared mechanism not only enhances the synergy within signaling pathways but also represents a potential alternative target for therapeutic interventions. Upon binding of IL-5 to IL-5R, a conformational change occurs, bringing the receptor into proximity with the c subunit, thus forming a stable complex. This interaction triggers the phosphorylation of the c subunit, which activates several signaling pathways, including JAK-STAT, MAPK, and PI3K (11). These pathways regulate key processes such as eosinophil proliferation, activation, chemotaxis, and survival.

IL-4 and IL-13, typical Th2 cytokines, synergize with IL-5 to promote eosinophil-mediated inflammatory responses, forming the core pathological mechanisms of allergic and eosinophil-associated diseases. IL-4 primarily stimulates B cells to produce IgE, which is pivotal in initiating and sustaining allergic reactions (17). In contrast, IL-13 directly induces structural and functional changes in the airway, including smooth muscle contraction, increased mucus secretion, and airway remodeling (18, 19). IL-5 plays a central role in promoting eosinophilia, activation, and tissue infiltration, making it a key mediator of eosinophil-associated inflammation (7, 8). Thus, IL-4, IL-13, and IL-5 each contribute to different aspects of the Th2 inflammatory response, together forming a complementary signaling network that regulates the pathological processes of allergies and eosinophil-associated diseases.

Both IL-4 and IL-13 activate the JAK/STAT signaling pathway via their respective receptors, IL-4R and IL-13R, while IL-5 activates the downstream JAK2/STAT5 axis. Upon binding to IL-5R, IL-5 rapidly induces JAK2 autophosphorylation and recruits STAT5 transcription factors to the receptor’s intracellular region, thereby forming the classical JAK2/STAT5 signaling axis (20). This pathway plays a central role in eosinophil survival, proliferation, and effector functions. However, IL-5 signaling is not limited to the STAT5 pathway. In THP-1-derived macrophages, IL-5 upregulates ABCA1 expression and promotes cholesterol efflux through the miR-211-mediated JAK2/STAT3 pathway, indicating a non-classical function in lipid metabolism regulation (20). Similarly, in a model of angiotensin II-induced cardiac remodeling, IL-5 exerts cardioprotective effects by inhibiting M2-type macrophage differentiation and activating the STAT3 pathway, whereas IL-5 deficiency results in increased fibrosis (21).

The activation of STAT5 is finely regulated. Conformational changes in the receptor complex influence the efficiency of JAK2 recruitment, while negative regulatory molecules, such as the Suppressor of Cytokine Signaling (SOCS) family, can target phosphorylated STAT5 for degradation, limiting the duration of signaling (22). In patients with severe eosinophilic asthma, the IL-5-driven eosinophil ‘priming’ state is closely associated with sustained STAT5 activation, explaining the effectiveness of anti-IL-5 therapy in suppressing the inflammatory response (22). This IL-5-driven priming lowers the activation threshold for other βc-family cytokines (such as IL-3 and GM-CSF) by upregulating the surface expression of the common β-receptor chain and maintaining intracellular signaling molecules in a pre-activated metastable state, thereby amplifying the inflammatory cascade.

When IL-5 binds to its receptor, it activates the downstream PI3K/AKT signaling pathway. In the type 2 inflammatory microenvironment, IL-5 does not act in isolation but forms a synergistic network with epithelial-derived alarmins, such as thymic stromal lymphopoietin (TSLP), IL-33, and the myeloid growth factor GM-CSF (23, 24). Epithelial cells release TSLP and IL-33 in response to allergen or injury-induced stimulation, which activates type 2 innate lymphoid cells (ILC2s). These cells secrete large amounts of IL-5 and IL-13, creating a positive feedback loop (23). The PI3K/AKT pathway plays a critical role in IL-33 activation of ILC2s and induction of IL-5 production. In eosinophilic asthma, IL-33 significantly enhances ILC2 activation in the lungs, leading to increased IL-5 and IL-13 production. This effect is mediated by the upregulation of PI3K and AKT protein expression within ILC2s. Inhibition of the PI3K/AKT pathway with specific inhibitors markedly reduced IL-5 and IL-13 production by ILC2s (25). This demonstrates that the PI3K/AKT pathway is a crucial link between upstream inflammatory signals (such as IL-33) and downstream effector cytokine (IL-5) production.

IL-5 also activates the Ras/Raf-ERK (MAPK) signaling pathway, which is involved in cell proliferation and differentiation, and plays a significant role in eosinophil activation (26). Binding of IL-5 to its receptor activates key kinases in the MAPK pathway. In human peripheral blood eosinophils, IL-5 stimulation induces rapid phosphorylation (activation) of p38 and ERK1/2 (27). This activation is essential for IL-5 signaling. For example, IL-5-induced phosphorylation of p38 and ERK1/2 correlates with increased mRNA levels of interleukin-1 (IL-1), suggesting that the MAPK pathway mediates IL-5 regulation of inflammatory gene expression (27). In obesity-associated asthma, IL-5 exacerbates inflammation through the p38 MAPK/NF-κB signaling pathway. Certain compounds, such as reticuline, have been shown to attenuate inflammation by inhibiting this pathway (28). Additionally, elevated phosphorylation levels of MAPK pathway members (p-ERK, p-JNK, p-p38), key inflammatory mediators, have been observed in lung tissues from a mouse model of allergic asthma and in airway epithelial cells stimulated by house dust mites (29).

## 3 Mechanisms of IL-5 in eosinophil-associated diseases

### 3.1 Role of IL-5 in asthma

#### 3.1.1 Promotion of eosinophil maturation and recruitment

IL-5 is a cytokine predominantly produced by Th2 cells that specifically promotes eosinophil maturation and differentiation (30). During the pathogenesis of asthma, IL-5 is released from activated Th2 cells and other sources, acting on eosinophil progenitor cells in the bone marrow to stimulate their proliferation and differentiation into mature eosinophils. Studies have demonstrated that IL-5 facilitates the differentiation of eosinophils by acting on their precursor cells in the bone marrow (2). This process is crucial in the chronic inflammatory response characteristic of asthma, as eosinophilic infiltration is a hallmark feature of the disease, closely linked to its severity and the risk of acute exacerbations.

In addition to promoting maturation, IL-5 plays a key role in eosinophil recruitment. It induces the release of eosinophils from the bone marrow into circulation, which further facilitates their migration to the airways and lung tissue (30). In asthmatic airways, IL-5 enhances eosinophil migration through synergy with other cytokines, such as IL-4 and IL-13 (23). This recruitment leads to localized eosinophil infiltration in the airways, which exacerbates inflammation and airway hyperresponsiveness.

#### 3.1.2 Enhanced eosinophil activation and inflammatory mediator release

IL-5 not only promotes eosinophil maturation and recruitment but is also directly involved in their activation. In the airways, eosinophils become activated upon IL-5 stimulation, leading to the release of various inflammatory mediators, such as eosinophil cationic protein (ECP) and IL-5 itself. These mediators further contribute to airway inflammation and tissue damage (3). Studies have shown that elevated levels of IL-5 are strongly associated with markers of eosinophil activation in the airways of asthma patients (31). This activation process is particularly pronounced during acute asthma exacerbations, highlighting the important role of IL-5 in the pathological progression of asthma.

### 3.2 Role of IL-5 in chronic obstructive pulmonary disease

#### 3.2.1 Association with the COPD eosinophil phenotype

Chronic Obstructive Pulmonary Disease (COPD) has traditionally been characterized as a predominantly neutrophilic inflammatory disorder. However, increasing evidence suggests that eosinophilic airway inflammation is present in approximately 20-40% of COPD patients (32). To facilitate precision management, clinical practice often employs blood eosinophil count (BEC) thresholds to stratify patients; specifically, BEC ≥150 cells/µL is used to identify those at risk for exacerbations, while BEC ≥300 cells/µL serves as a critical cutoff for predicting a favorable response to inhaled corticosteroids and anti-IL-5 biologics (33).This subset, referred to as the “eosinophilic phenotype” of COPD, is closely associated with the key cytokine interleukin-5 (IL-5). The eosinophilic phenotype of COPD exhibits distinct clinical and biological features. Unlike asthma, where eosinophils in circulation are more abundant, COPD patients display a significantly lower proportion of inflammatory eosinophils (iEos) in their blood compared to asthma patients, smokers without COPD, and healthy individuals (34). This discrepancy may explain the differing therapeutic efficacy of IL-5-targeting treatments in asthma and COPD (12). Moreover, the eosinophilic phenotype is linked to more pronounced airway remodeling (14) and may predict a better response to corticosteroid therapy (15).

#### 3.2.2 Effect on acute exacerbations of COPD

IL-5 plays a central role in regulating type 2 inflammatory responses, which are especially critical in acute exacerbations of COPD. In COPD patients, elevated IL-5 expression is strongly associated with eosinophil infiltration into the airways and lung tissue. This eosinophilic phenotype not only exacerbates inflammation but also contributes to alveolar cell death and the development of emphysema (16). RNA sequencing analysis has shown that IL-5-mediated signaling pathways activate the alternative activation of myeloid cells and macrophages, further amplifying the inflammatory response and tissue damage (20).

Research has also indicated that in the peripheral blood of patients with acute exacerbations of COPD (AECOPD), both the proportion of Th2 cells and serum IL-4 levels are significantly elevated, along with an increase in the number of ILC2s (21). These ILC2s exhibit high expression of GATA3, ROR, and CRTH2 mRNA and show significantly upregulated MHC II and CD80 molecules on their surface. More importantly, the proportion of MHC II+ ILC2s was positively correlated with the proportion of Th2 cells in the peripheral blood of AECOPD patients. This suggests that ILC2s may enhance Th2-type adaptive immune responses via their antigen-presenting functions. *In vitro* co-culture experiments confirmed that ILC2s isolated from AECOPD patients significantly promoted the differentiation of CD4+ T cells into Th2 cells and increased the production of Th2-associated cytokines such as IL-4, IL-5, and IL-13. This effect could be inhibited by anti-MHC II antibodies (22).

In addition to ILC2s, eosinophils themselves are critical target cells for IL-5. In virus-induced AECOPD, patients’ serum levels of soluble IL-5 receptor (sIL-5R) are significantly elevated, and changes in its concentration correlate with improvements in lung function (FEV1) (24). This suggests that activation of the IL-5 signaling pathway is closely associated with the clinical course of AECOPD. The role of IL-5 in acute exacerbations of COPD primarily involves driving a specific inflammatory endotype—eosinophil/T2 inflammatory exacerbations (35). By activating ILC2s and eosinophils, IL-5 amplifies the Th2-type immune response and contributes to the induction of acute exacerbations.

### 3.3 Role of IL-5 in eosinophilic gastroenteritis

Eosinophilic Gastroenteritis (EGE) and related Eosinophilic Gastrointestinal Disorders (EGIDs) are a group of gastrointestinal diseases characterized by abnormal eosinophil infiltration into gastrointestinal tissues, leading to chronic inflammation (36). IL-5, primarily produced by activated type 2 helper T cells (Th2 cells), is the key cytokine regulating eosinophil production, activation, survival, and tissue recruitment (37). In EGE, an aberrant immune response results in elevated IL-5 levels, triggering a cascade of pathological processes.

IL-5 specifically promotes the proliferation, differentiation, and maturation of eosinophils in the bone marrow, significantly prolonging the survival of mature eosinophils in the bloodstream and tissues (38). Elevated IL-5 levels in patient serum have been shown to correlate with disease activity, suggesting that IL-5 may serve as a potential biomarker for monitoring disease progression (39). Additionally, IL-5 works synergistically with eosinophil chemokines, such as eosinophil-activated chemokines, to direct eosinophil migration from the circulation to the gastrointestinal mucosa (40). This infiltration can affect any level of the gastrointestinal tract, from the mucosal to the transmural layers, and even the plasma membrane layer, leading to a variety of clinical symptoms (37).

**Figure 1:**
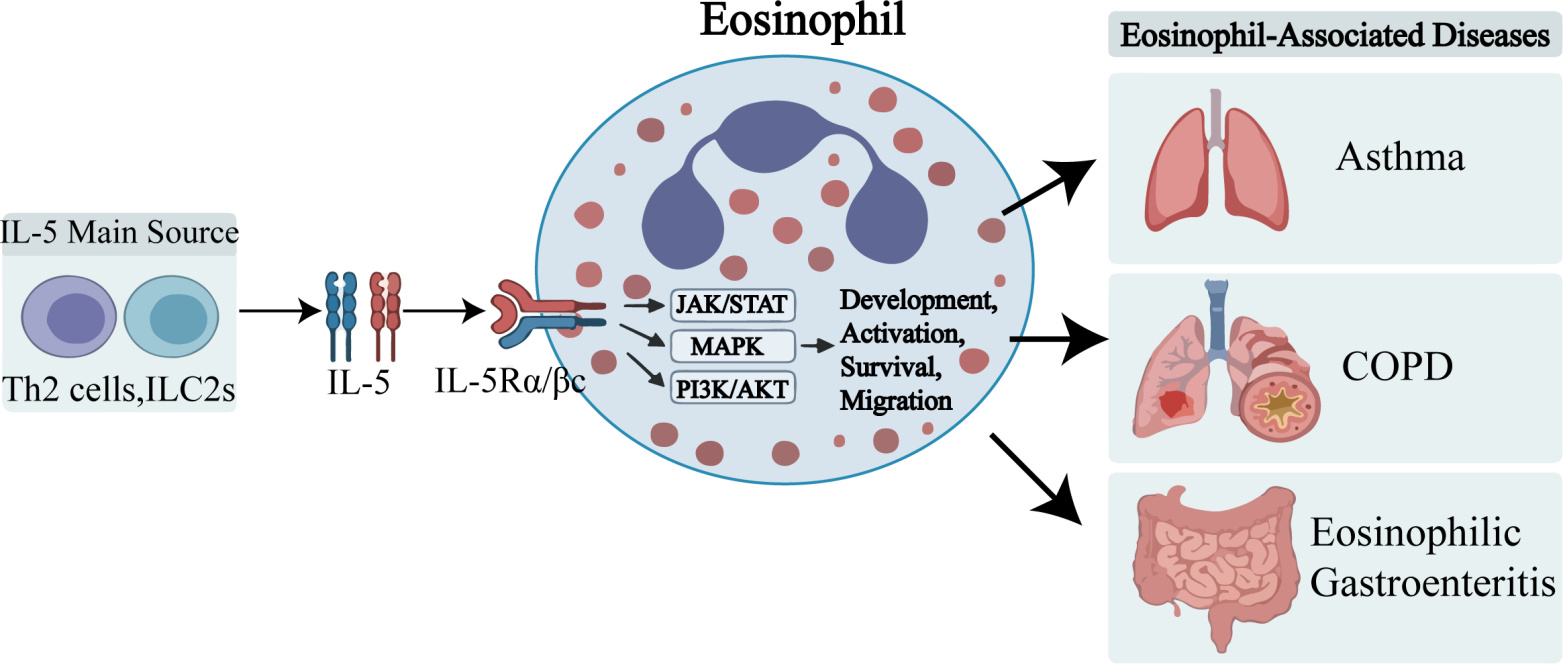
Central role of the IL-5 axis in eosinophil-associated diseases and therapeutic interventions. Interleukin-5 (IL-5) is primarily secreted by Type 2 helper T (Th2) cells and Group 2 innate lymphoid cells (ILC2s). Upon binding to its receptor complex, which consists of the IL-5 receptor alpha subunit (IL-5R) and the common subunit (c), IL-5 triggers intracellular signaling cascades, including the JAK/STAT, MAPK, and PI3K/Akt pathways. These signaling events promote the development, activation, survival, and migration of eosinophils. Excessive eosinophilic inflammation is a key pathological driver in several diseases, including asthma, chronic obstructive pulmonary disease (COPD), and eosinophilic gastroenteritis.

Studies have indicated that the food antigen-specific Th2 cell response in EGE patients is unique, with a large subpopulation of IL-5-producing Th2 cells (IL-5+ Th2 cells) (37). This immune response pattern contrasts with that of classical food allergies (e.g., peanut allergy), which are predominantly IgE-mediated and typically involve IL-5-negative Th2 cells (IL-5- Th2 cells) (37). This distinction helps explain why eosinophilic gastroenteritis primarily presents with eosinophil-mediated tissue damage rather than rapid allergic reactions such as tachyphylaxis. See **Figure 1** for more details.

## 4 Targeted therapy against interleukin-5

### 4.1 Anti-IL-5 monoclonal antibodies

#### 4.1.1 Molecular design rationale and pharmacological advantages of mepolizumab

Targeting IL-5, rather than directly blocking its receptor, offers a selective approach to suppress pathological inflammatory responses while maintaining immune system homeostasis. Mepolizumab, a humanized anti-IL-5 monoclonal antibody, is specifically designed to neutralize IL-5 rather than targeting the IL-5 receptor α subunit (IL-5Rα) (41). This design is based on strong biological rationale: IL-5, a key cytokine responsible for eosinophil differentiation, activation, and survival, is primarily secreted by T helper 2 (Th2) cells and group 2 innate lymphoid cells (ILC2s). It is overexpressed in eosinophil-associated disorders, including severe eosinophilic asthma (SEA), eosinophilic granulomatosis with polyangiitis (EGPA), and hypereosinophilic syndrome (HES) (42). By sequestering free IL-5, mepolizumab prevents its binding to the IL-5Rα/βc heterodimer, thereby inhibiting activation of the downstream Janus kinase-signal transducer and activator of transcription (JAK-STAT) signaling pathway. This mechanism reduces eosinophil egress from the bone marrow and induces apoptosis of these cells in peripheral tissues.

In contrast to IL-5R-targeting strategies, such as benralizumab, mepolizumab avoids off-target damage to other IL-5Rα-expressing immune cells (e.g., basophils and B-cell subsets), thus preserving immune system homeostasis (43). Clinical studies have shown that mepolizumab reduces peripheral eosinophil counts by over 80%, while maintaining a basal population of residual eosinophils with a phenotype similar to that of healthy controls. These findings suggest that IL-5 mainly regulates eosinophil expansion during inflammation, rather than being essential for eosinophil development (44). This selective targeting not only controls pathological inflammation but also preserves the physiological functions of eosinophils in tissue repair, antiparasitic immunity, and antiviral defense (45).

The humanized IgG1κ isotype structure is a key pharmaceutical innovation, granting mepolizumab high antigen affinity and low immunogenicity. Engineered on a humanized IgG1κ backbone, with variable regions derived from the murine anti-IL-5 antibody SCH55700, mepolizumab incorporates murine complementarity-determining regions (CDRs) into the human IgG1 framework, resulting in less than 5% non-human sequence content. This design significantly reduces immunogenicity risk and the incidence of anti-drug antibodies (ADAs) in clinical trials (46). The antigen-binding fragment (Fab) of mepolizumab exhibits an affinity constant (Kd) for IL-5 in the 10^-10^ M range, ensuring efficient neutralization of circulating IL-5 and blockade of receptor binding at picomolar concentrations. Despite its IgG1 subclass designation, mepolizumab undergoes Fc-region engineering to eliminate its ability to activate complement-dependent cytotoxicity (CDC) and antibody-dependent cell-mediated cytotoxicity (ADCC), preventing damage to normal IL-5R-expressing cells (46). The “high-affinity neutralization/low effector function” design ensures that the drug acts exclusively through cytokine neutralization, rather than target cell depletion, thereby enhancing its safety profile for long-term clinical use. Long-term studies have shown that 36 months of mepolizumab treatment reveal no novel safety signals, with stable renal function, liver enzyme levels, and infection rates in treated patients (47). This structural design also confers excellent solution stability and tolerability for subcutaneous injection, providing a robust foundation for managing chronic diseases.

The pharmacokinetic (PK) profile of mepolizumab aligns with the therapeutic needs of chronic airway and systemic eosinophilic disorders, supporting long-acting subcutaneous administration regimens. This approach ensures sustained clinical efficacy and improved patient compliance. After a single 100 mg subcutaneous injection, mepolizumab reaches peak plasma concentration (Tmax) within 4–8 days, with an average bioavailability of approximately 80%. Steady-state trough concentrations are achieved by week 12, and the elimination half-life (t½) ranges from 20 to 26 days. Mepolizumab is primarily metabolized via non-specific proteolytic pathways, with clearance independent of hepatic or renal function, eliminating the need for dosage adjustment in patients with hepatic or renal impairment. These PK characteristics support a fixed-dose regimen of once every 4 weeks to maintain a stable IL-5-neutralizing concentration and suppress eosinophil regeneration effectively. Real-world data have shown that this dosing schedule yields a compliance rate of over 90%, significantly higher than that of daily inhaled or oral therapies (47).

Importantly, long-acting administration enables prolonged inhibition of the IL-5 signaling pathway, blocking eosinophil-mediated tissue remodeling. Bronchial biopsy studies in severe asthma patients have shown that 24 weeks of mepolizumab treatment reduce eosinophil infiltration in the airway submucosa by 76%, accompanied by a significant decrease in serum profibrotic factor levels. These findings suggest that mepolizumab not only controls acute inflammation but may also slow airway remodeling (48). This strong pharmacokinetic-pharmacodynamic (PK-PD) concordance makes mepolizumab an ideal candidate for disease-modifying therapy in eosinophil-associated disorders. Additionally, reslizumab has emerged as another important therapeutic option for these diseases, owing to its distinct molecular design and functional properties.

#### 4.1.2 Mechanism and clinical efficacy of reslizumab and benralizumab

Reslizumab is a humanized anti-interleukin-5 (IL-5) monoclonal antibody designed with an IgG4gnedalleu conferring unique molecular engineering advantages (47). Following the engineering optimization of its Fab fragment, reslizumab exhibits a picomolar affinity (KD ≈ 80 pM) for the free human IL-5 ligand. It efficiently binds to the IL-5 ligand, thereby blocking its interaction with the IL-5Rα receptor on the surface of eosinophils, which subsequently inhibits downstream signal transduction. The selection of the IgG4 isotype precludes Fc-mediated complement activation and antibody-dependent cellular cytotoxicity (ADCC), thus mitigating non-specific inflammatory responses and enhancing therapeutic safety (48). Furthermore, the IgG4 molecule has a prolonged *in vivo* half-life (approximately 24 days), providing feasibility for an intravenous administration regimen every 4 weeks. This facilitates the maintenance of stable drug concentrations and sustained IL-5 neutralization (49). Clinical studies have demonstrated that in patients with severe eosinophilic asthma treated with reslizumab, the mean reduction in peripheral blood eosinophil counts exceeds 90%, accompanied by significant improvements in lung function and a substantial reduction in the annualized exacerbation rate (a decrease of >50%) (50). Leveraging its high specificity and long-acting ligand neutralization capabilities, reslizumab has emerged as a crucial option for precise intervention in the IL-5 pathway, particularly suitable for patient populations with sputum eosinophils ≥ 3% or peripheral blood eosinophilsy,nd cells/ph (51). Although the intravenous route of administration limits its clinical utility to some extent, the drug’s advantages in rapid onset of action and the inhibition of eosinophil survival confer irreplaceable clinical value in the prevention of acute exacerbations and corticosteroid-sparing strategies.

In contrast to the IL-5 ligand-targeted reslizumab, benralizumab, a monoclonal antibody targeting the IL-5Rα receptor, possesses the advantage of a dual mechanism of action: it not only blocks the binding of IL-5 to IL-5Rα but also directly depletes IL-5Rα-expressing eosinophils via Fc-mediated ADCC (52). This property enables it to demonstrate a profound eosinophil-depleting effect in diseases such as severe eosinophilic asthma, chronic rhinosinusitis with nasal polyps (CRSwNP), and eosinophilic granulomatosis with polyangiitis (EGPA) (53). Clinical study results indicate that benralizumab can suppress peripheral blood eosinophils to below the limit of detection within hours, and this effect can be sustained for several weeks, significantly outperforming ligand-blocking agents in the directness of cell depletion (54). Furthermore, in patients with allergic bronchopulmonary aspergillosis (ABPA), the drug effectively reduces the frequency of acute exacerbations, decreases the required dosage of oral corticosteroids, and improves lung function, with particularly pronounced efficacy in patients presenting with mucus plugs (55). Notably, IL-5Rα is expressed not only on the surface of mature eosinophils but also on their bone marrow progenitors, as well as on certain epithelial cells and B-cell subsets (21). The depleting effect of benralizumab on progenitor cells holds the potential to inhibit eosinophil generation at the source, which provides a plausible mechanistic rationale for the durability of its long-term efficacy. A meta-analysis targeting patients with chronic obstructive pulmonary disease (COPD) with an eosinophilic phenotype confirmed that anti-IL-5/IL-5Rα therapies can significantly reduce the annualized exacerbation rate and the incidence of serious adverse events (56). The aforementioned data collectively indicate that IL-5Rα possesses distinct potential for deeper biological intervention compared to the IL-5 ligand itself. Among IL-5Rα-targeted biologic agents, benralizumab, through its unique molecular design, achieves a “dual-inhibition” effect by blocking IL-5 signal transduction and directly inducing the apoptosis of eosinophils and basophils.

#### 4.1.3 The “dual inhibition” effect of benralizumab

Benralizumab is a humanized IgG1κ monoclonal antibody that offers a core advantage through its ability to target IL-5Rα while retaining FcγRIIIa (CD16a)-binding capacity via Fc-region engineering. This design facilitates the efficient activation of effector cells, such as natural killer (NK) cells and macrophages, thereby enhancing antibody-dependent cell-mediated cytotoxicity (ADCC) (49). This mechanism not only blocks IL-5 signal transduction but also directly induces apoptosis of eosinophils and basophils, resulting in a “dual inhibition” effect (50). Unlike most IgG4-based biologics used in chronic inflammatory diseases, the IgG1 subclass inherently possesses potent effector functions. Studies confirm that, while the Fc region of benralizumab does not undergo defucosylation, its spatial conformation effectively recruits CD16a-expressing immune cells. These cells form immune synapses on the eosinophil surface, triggering the release of granzyme B and perforin, which ultimately induce target cell lysis (51).

Benralizumab not only targets circulating mature eosinophils but also penetrates the bone marrow microenvironment, depleting IL-5Rα-expressing eosinophil progenitor cells (EoPs) and precursors. This direct elimination of eosinophil precursors via ADCC effectively “resets” eosinophil production at the source (52). Additionally, IL-5Rα is expressed at low levels on basophils, mast cell subsets, and memory T-cell populations. By targeting this receptor, benralizumab may exert broader immunomodulatory effects, providing an additional mechanistic basis for its potent anti-inflammatory activity.

Benralizumab exerts systemic anti-inflammatory effects by depleting both circulating and tissue-resident eosinophil pools. It eliminates mature IL-5Rα-expressing eosinophils in peripheral blood via ADCC, while blocking IL-5Rα signaling to inhibit the terminal differentiation of eosinophil precursors in the bone marrow (53). This action attenuates IL-5-mediated survival signals in the tissue microenvironment, promoting apoptosis of tissue-infiltrating eosinophils. A peripheral eosinophil count >300/μL during benralizumab treatment has been identified as a predictive biomarker of therapeutic efficacy (54). Clinical trials and data confirm that benralizumab is more effective and safer than placebo in patients with severe eosinophilic asthma (SEA) (51, 55, 56), and it has proven effective in reducing oral corticosteroid (OCS) requirements (57).A real-world study of 1002 adult SEA patients, followed for 48 weeks, demonstrated that benralizumab significantly improves health-related quality of life, reduces the incidence of exacerbations, enhances lung function, and decreases OCS use (58). These findings further substantiate the clinical value of IL-5Rα-targeting therapies. Ongoing research into innovative strategies, such as multi-target synergistic interventions, broad-spectrum inhibition of downstream signaling pathways, and source-specific molecular silencing, is expanding the landscape of precision treatments for eosinophil-associated disorders.

### 4.2 Novel strategies for targeting the IL-5 signaling pathway

#### 4.2.1 Bispecific antibody design

Simultaneous Targeting of IL-5 and Upstream Initiating Cytokines. Type 2 inflammation is driven by a multi-cytokine network, and single-target inhibition often fails to fully control the complex disease phenotypes (59). Recent advances in bispecific antibody (BsAb) technology have enabled the concurrent blockade of IL-5 and other key cytokines (e.g., thymic stromal lymphopoietin (TSLP), IL-4, IL-13) (59). For example, TSLP/IL-5 bispecific antibodies can inhibit both ILC2 activation mediated by epithelial alarmin release and subsequent eosinophil expansion. Multi-target strategies have been successfully applied in multiple myeloma treatment; for instance, BCMA/GPRC5D bispecific antibodies have received regulatory approval for clinical use (60). Although no IL-5-related bispecific antibodies have entered clinical development yet, the high-affinity nanobody AIL-A96-Fc—identified through phage display screening of an alpaca antibody library—has been shown to effectively block IL-5 binding to IL-5Rα. It also exhibits cross-reactivity between humans and non-human primates, laying the groundwork for the development of inhaled formulations or multi-target biologics (61). Additionally, a novel humanized anti-IL-5 Fab fragment (20A12-Fab-H12L3), with a Kd of 1.236×10^-9^ M for IL-5, effectively inhibits IL-5-induced cell proliferation and is suitable for inhaled administration, allowing for high-concentration drug accumulation in local tissues with minimal systemic exposure (61).

#### 4.2.2 Small-molecule inhibitors

Broad-Spectrum Inhibition of Downstream JAK1/2 and STAT5 Signaling. Small-molecule kinase inhibitors have emerged as important complements to biologics due to their oral bioavailability and cost-effectiveness. Selective JAK1/2 or STAT5 inhibitors can broadly inhibit multiple γc family cytokines, including IL-5, IL-3, and granulocyte-macrophage colony-stimulating factor (GM-CSF) (62). Studies have confirmed that JAK inhibitors significantly reduce serum immunoglobulin E (IgE) levels, eosinophil counts, and Th2-type cytokine levels in type 2 inflammatory diseases such as asthma and atopic dermatitis (62). While no JAK inhibitors specifically targeting the IL-5 pathway have been approved for clinical use, exploratory studies of baricitinib—a JAK1/2 inhibitor—have shown preliminary efficacy in refractory eosinophilic esophagitis (62). Additionally, protein-protein interaction inhibitors targeting STAT5 are in early preclinical development. These agents aim to block STAT5 dimerization and nuclear translocation, inhibiting the transcriptional activation of downstream pro-survival genes (e.g., Bcl-xL, Mcl-1) (63). Despite the challenges in selective modulation, advances in structural biology have identified a small-molecule binding pocket within the Src homology 2 (SH2) domain of STAT5, providing essential theoretical support for rational drug design (64).

#### 4.2.3 IL-5 mRNA-targeting technologies

Source-Specific Silencing with siRNA and Antisense Oligonucleotides. RNA interference (RNAi) technology offers a novel strategy for silencing IL-5 expression at the post-transcriptional level, enabling source-specific inhibition (65). Small interfering RNAs (siRNAs), delivered via lipid nanoparticles (LNPs) or N-acetylgalactosamine (GalNAc)-conjugated systems, can efficiently target IL-5 mRNA in hepatocytes or immune cells, providing long-term inhibitory effects. Although no IL-5-specific siRNAs have entered clinical trials, analogous technologies have been successfully implemented in clinical practice for lipid-lowering therapies (e.g., inclisiran targeting PCSK9) and antiviral treatments, validating the feasibility of this approach (66). Antisense oligonucleotides (ASOs) inhibit IL-5 synthesis through ribonuclease H (RNase H)-mediated mRNA degradation. ASOs offer several advantages, including prolonged action and independence from protein target conformation, making them particularly suitable for local microenvironments where antibody-mediated ligand neutralization is less effective.

#### 4.2.4 Targeting the common βc subunit

**Figure 2:**
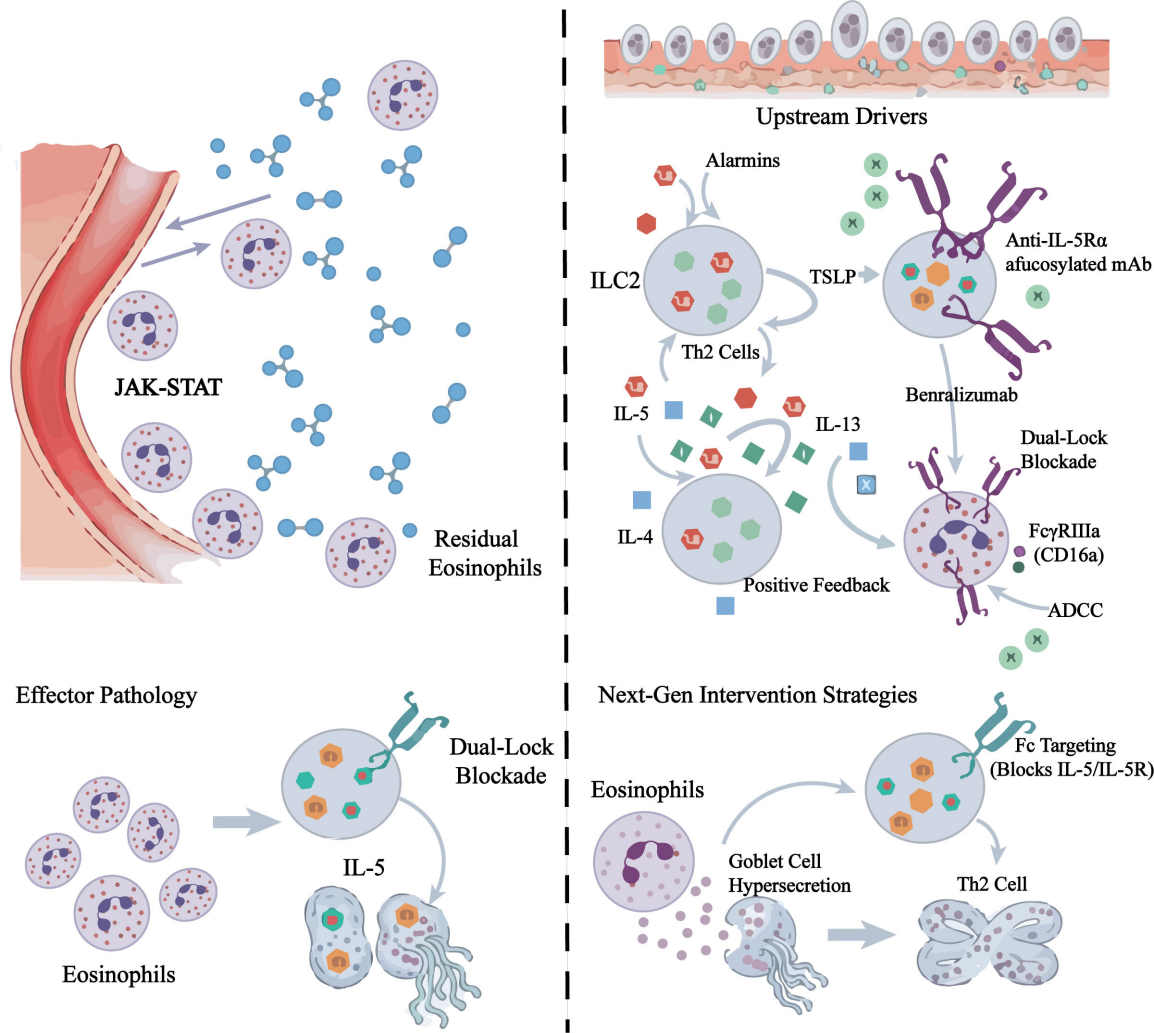
Pharmacological mechanisms of Anti-IL-5/IL-5Rα biologics and emerging multi-target strategies. Ligand-targeting monoclonal antibodies (e.g., Mepolizumab, Reslizumab) neutralize soluble IL-5 homodimers, preventing their binding to the IL-5Rα/βc complex and subsequent JAK2/STAT5 activation. This approach selectively inhibits the expansion of inflammatory eosinophils while preserving residual homeostatic eosinophils. The anti-IL-5Rα antibody (Benralizumab) directly blocks the receptor and triggers antibody-dependent cellular cytotoxicity (ADCC) mediated by NK cells via FcγRIIIa engagement, leading to near-complete depletion of eosinophils and their bone marrow precursors. Future precision medicine approaches aim to incorporate multi-target interventions, such as bispecific antibodies (BsAbs) targeting TSLP/IL-5 or IL-4/IL-5, and small-molecule JAK1/2 inhibitors, to counteract the redundancy and synergy within the type 2 inflammatory network (e.g., IL-33, GM-CSF, and TSLP).

Simultaneous Interference with IL-3/GM-CSF/IL-5 Signaling Pathways. The βc subunit is a shared signaling component for IL-3, IL-5, and GM-CSF, making it an ideal target for multi-pathway synergistic inhibition (67). Monoclonal antibodies or small-molecule compounds targeting βc can concurrently attenuate the activation of eosinophils, neutrophils, and the monocyte-macrophage system, offering unique application potential in complex inflammatory diseases. Animal studies have shown that βc-deficient mice exhibit resistance to allergen-induced airway inflammation (68). However, the βc subunit is widely involved in hematopoietic homeostasis, and complete blockade of βc function could result in myelosuppression and other safety risks. Therefore, the development of partial antagonists or tissue-targeted delivery systems is necessary to balance therapeutic efficacy with safety (68). Although no βc-targeted drugs are currently in clinical development, their theoretical potential has spurred exploration of innovative approaches, including allosteric inhibitors and conditionally activated antibodies. See **Figure 2** for more details.

## 5 Combined targeting of IL-5 and other cytokines

### 5.1 Combination targeting of IL-5 and other cytokines

#### 5.1.1 Combination targeting of IL-5 and IL-4/IL-13

Studies have shown that single-target inhibition is often insufficient to fully block the complex Th2 inflammatory pathway, leading to suboptimal responses or inadequate disease control in some patients (69). In light of this, combination therapies targeting IL-5 and IL-4/IL-13 have emerged as a strategy to more comprehensively suppress Th2-mediated immune responses through the synergistic action of multiple targets, reducing inflammation and remodeling in the airways and tissues.

Current clinical studies and trials have demonstrated that bispecific antibodies and combination monoclonal antibody treatments perform well in terms of both safety and efficacy, showing significant clinical benefit potential. For example, a retrospective study reported the safety of a combination therapy involving multiple biologics, including antibodies targeting IgE, IL-5/IL-5R, and IL-4/IL-13. No serious adverse events were observed among patients, most of whom were treated for asthma and related conditions, suggesting that combined biologic therapy is both well-tolerated and effective (7). Additionally, case reports have highlighted that sequential or combined use of anti-IL-5 receptor and anti-IL-4/13 receptor monoclonal antibodies can achieve comprehensive control of complex eosinophilic diseases, addressing the limitations of monotherapy (70).

To make this therapeutic prospect more concrete, novel bispecific molecules are currently advancing in preclinical and clinical development. A prominent example is BBT002, a novel tetravalent “2 + 2” bispecific antibody engineered to simultaneously target IL-4Rα (thereby blocking both the IL-4 and IL-13 pathways) and IL-5. Recent preclinical evaluations demonstrate that BBT002 effectively inhibits IL-4, IL-13, and IL-5 signal transduction pathways concurrently without a loss of potency. In murine asthma models, BBT002 significantly outperformed single-target therapies (such as mepolizumab and dupilumab analogues) by markedly reducing eosinophils in alveolar lavage fluid, completely suppressing total serum IgE levels, and substantially alleviating bronchial inflammatory cell infiltration and mucus production (71). Research indicates that combination targeting can simultaneously block multiple signaling pathways, effectively reducing the infiltration of inflammatory cells and the levels of inflammatory factors, thereby alleviating tissue damage and remodeling, resulting in better therapeutic outcomes. In summary, the combination targeting of IL-5 and IL-4/IL-13 takes advantage of their synergistic effects in the Th2 inflammatory response to more effectively and comprehensively suppress eosinophil-associated inflammation, reducing airway and tissue inflammation and remodeling. This strategy offers new hope and therapeutic options for the treatment of asthma, allergic rhinitis, and other eosinophil-mediated diseases. As bispecific antibodies like BBT002 and combination monoclonal antibody therapies undergo further clinical validation, their potential for broader application will continue to expand.

#### 5.1.2 Combination targeting of IL-5 and IL-33/ST2 pathway

IL-33, as a key alarmin, is primarily released by damaged cells and activates immune cells, particularly Th2 cells and eosinophils, through its receptor ST2, promoting the release of inflammatory mediators. The IL-33/ST2 pathway plays a central role in various inflammatory diseases, especially those associated with eosinophils, such as asthma, allergic rhinitis, and certain parasitic infections, where it exhibits significant immunoregulatory functions (72). Given the close association between the IL-33/ST2 and IL-5 pathways, the combined inhibition of these two signaling pathways is considered a promising therapeutic strategy. For instance, the monoclonal antibody targeting IL-33, torudokimab, has shown high efficacy in neutralizing IL-33 activity in preclinical studies, demonstrating strong therapeutic potential (73). Additionally, IL-5-targeting antibodies such as mepolizumab and reslizumab have been approved for the treatment of severe eosinophilic asthma. The combination or simultaneous inhibition of the IL-33/ST2 and IL-5 pathways is expected to yield superior therapeutic outcomes. Currently, research on combination antibody therapies is actively progressing, with preliminary data supporting their potential application in eosinophil-associated diseases such as asthma. Animal model studies indicate that combined inhibition of the IL-33/ST2 and IL-5 pathways significantly reduces ILC2 activity and eosinophil infiltration, effectively alleviating airway inflammation and hyperreactivity (74, 75). Moreover, traditional Chinese medicine formulas such as modified Yupingfeng San have been found to modulate the IL-33/ST2 pathway, reducing IL-5 and other Th2 cytokine levels, thereby improving symptoms of allergic rhinitis. This suggests that multi-target combination therapeutic strategies hold broad preclinical prospects.

### 5.2 Application strategies of multi-target therapy in different diseases

#### 5.2.1 Multi-target combination therapy in asthma treatment

In the treatment of severe asthma, targeted therapy solely focused on IL-5 has shown clear efficacy in reducing eosinophil-mediated inflammation; however, its clinical effectiveness remains somewhat limited. In some severe cases, blocking the IL-5 pathway alone is insufficient to fully control the disease, suggesting that the pathogenesis of asthma involves complex interactions among multiple inflammatory pathways. In response to this phenomenon, multi-target combination therapies have increasingly gained attention. The anti-IgE monoclonal antibody omalizumab exerts its effect by binding to free IgE in the circulation, blocking its interaction with mast cells and basophils, thereby preventing their activation and the release of inflammatory mediators. This mechanism allows for control of allergic inflammation through a different pathway. Combining omalizumab with anti-IL-5 therapy enhances the overall anti-inflammatory effect, making it particularly suitable for severe asthma patients with elevated IgE levels and eosinophilic inflammation (76). Clinical case reports indicate that the combination of omalizumab and mepolizumab significantly improves asthma control, reduces acute exacerbations, and decreases the use of oral corticosteroids, with no major safety concerns (77). Furthermore, the combination of JAK inhibitors with anti-IL-5 therapy can block multiple cytokine signals, regulating the activation and secretion of immune cells, thereby more comprehensively suppressing airway inflammation and immune responses. Research has shown that JAK inhibitors can restore corticosteroid sensitivity in some steroid-resistant patients, reduce ILC2 cell proliferation, and decrease the release of inflammatory mediators, making them a promising adjunct to anti-IL-5 therapy (78).

The advantage of multi-target combination therapy lies in its synergistic inhibition of multiple key nodes in the pathological process of asthma, enabling more effective control of airway inflammation, reduction in acute exacerbations, and improvement in lung function and patient quality of life. Real-world studies have shown that combination therapy not only enhances clinical efficacy but also reduces dependence on oral corticosteroids, thereby alleviating the disease burden (79). In the future, with a deeper understanding of the immune pathogenesis of asthma, multi-target combination therapy based on individualized inflammatory phenotypes is expected to become the new standard for managing severe asthma, improving disease remission rates and patient quality of life.

#### 5.2.2 Multi-target exploration in the treatment of eosinophilic gastroenteritis

The traditional treatment for eosinophilic gastrointestinal disease (EGE) primarily relies on systemic corticosteroids and dietary therapy. However, long-term use of these treatments may lead to side effects, and some patients do not respond well, making multi-target combination therapy a growing area of research. In addition to targeting IL-5 to reduce the activation and survival of eosinophils, modulating the gut microbiota has shown significant potential in improving the gastrointestinal immune microenvironment. Dysbiosis is often associated with enhanced local inflammatory responses, and probiotics and other modulators can restore microbial balance, reduce the expression of inflammatory factors, and thereby enhance the efficacy of anti-IL-5 therapy. Studies have shown that probiotics such as Limosilactobacillus reuteri VHProbi^®^ M07 can significantly reduce Th2 cytokines like IL-5 and IL-13 in asthma models, decreasing inflammatory cell infiltration, suggesting their role in regulating both systemic and local immune responses (80). Additionally, drugs targeting gastrointestinal mucosal repair, such as natural medicines or biologics that promote mucosal healing and possess anti-inflammatory properties, have also been shown to effectively alleviate gastrointestinal symptoms (81). Clinical case reports indicate that the combination of the anti-IL-5 monoclonal antibody mepolizumab with other anti-allergic drugs, such as leukotriene receptor antagonists like montelukast, or corticosteroids, can achieve symptom relief and reduction in tissue inflammation, suggesting that multi-target treatment strategies can have complementary effects and improve therapeutic outcomes (82). The combined therapeutic approach of regulating the gut microbiota and targeting immune inflammation not only helps alleviate local inflammatory responses but also improves gut barrier function, reduces immune activation, and enhances the efficacy of anti-IL-5 treatment. Furthermore, factors such as psychological stress, which exacerbate intestinal inflammation through the CRH-mast cell axis, suggest that regulating neuroendocrine pathways or combining anti-inflammatory therapies may be promising future directions for research (83). Thus, multi-target treatment strategies, by simultaneously regulating immune cell activity, gut microbiota, and mucosal repair, provide a new approach for the treatment of eosinophilic gastrointestinal disease, offering more precise and individualized treatment options for clinical management of this condition. Further clinical trials are needed to verify their long-term efficacy and safety.

#### 5.2.3 Prospects of multi-target therapy in COPD treatment

Studies have shown that in COPD patients, besides neutrophils, an increase in eosinophils is closely associated with acute exacerbations and a decline in lung function. This is accompanied by elevated levels of inflammatory factors such as IL-1 receptor-like protein 1 and TNF-α, reflecting a complex inflammatory network (84, 85). These findings suggest that single-target inhibition may be insufficient to fully control the inflammatory process in COPD. Consequently, multi-target combination therapy has emerged as a promising strategy for COPD treatment. The combined inhibition of IL-5 with cytokines such as TNF-α and IL-17 can more effectively reduce airway inflammation, inhibit airway remodeling, and delay the decline in lung function. Clinical trials have shown that while anti-IL-5 monoclonal antibodies (e.g., mepolizumab) can reduce exacerbation frequency in eosinophilic COPD patients, their impact on lung function and quality of life is limited, indicating that blocking IL-5 alone may not be sufficient to fully improve the disease (86). Therefore, the combined inhibition of other key inflammatory factors, especially TNF-α and IL-17, is expected to complement IL-5-targeted therapy, yielding a synergistic effect for better therapeutic outcomes.

Additionally, drugs targeting oxidative stress and protease-antiprotease imbalance mechanisms (such as antioxidants and protease inhibitors) used in combination with anti-IL-5 therapy may effectively improve the pathological state of COPD. Research indicates that oxidative stress levels are closely related to the inflammatory status in COPD patients, and increased oxidative stress can promote the activation of eosinophils and the release of inflammatory mediators (87). Thus, combined antioxidant treatment and IL-5-targeted therapy may synergistically reduce the inflammatory burden in the airways and improve clinical outcomes.

**Figure 3:**
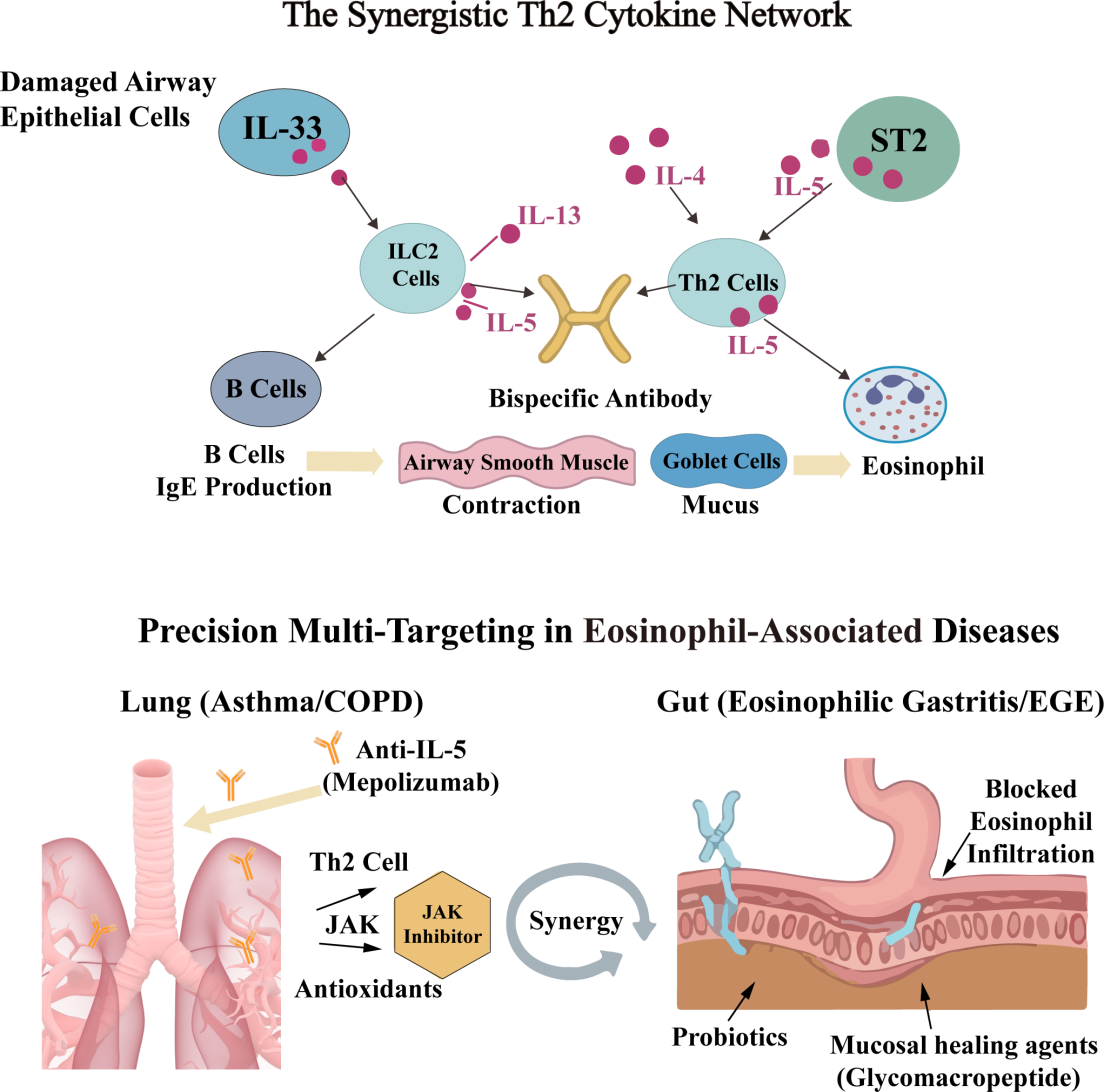
Synergistic cytokine networks and disease-specific multi-target strategies. Schematic of the “Alarmin-ILC2-IL-5” axis where IL-33 triggers a cascade of IL-4, IL-5, and IL-13, forming a positive feedback loop that drives chronic eosinophilic inflammation. Combined blockade aims to interrupt IgE production, mucus secretion, and cell recruitment simultaneously. Integrated management of eosinophilic disorders across organs. In pulmonary diseases (Asthma/COPD), precision therapy combines IL-5 inhibition with JAK/STAT blockade and anti-oxidative measures. In EGE, a tripartite strategy involving IL-5 neutralization, microbiota modulation by probiotics, and mucosal healing agents (e.g., Glycomacropeptide) is employed to restore gastrointestinal homeostasis and barrier integrity.

With the development of precision medicine, multi-target combination therapy provides more individualized and precise treatment strategies for COPD patients. By detecting biomarkers such as blood eosinophil count, it is possible to identify patients with different inflammatory phenotypes and design targeted combination therapy plans. For example, patients with blood eosinophil counts ≥300 cells/μL are more likely to benefit from anti-IL-5 therapy (88, 89). Furthermore, evaluating TNF-α and IL-17 levels, as well as oxidative stress markers, can systematically guide the combined treatment regimen and optimize therapeutic effects. Overall, the multi-target treatment strategy for COPD should not only include the IL-5 pathway but also address inflammatory factors such as TNF-α, IL-17, as well as the regulation of oxidative stress and protease-antiprotease balance. Future research should focus on assessing the safety and efficacy of multi-target combination therapy, exploring individualized treatment plans guided by biomarkers, with the aim of improving the prognosis and quality of life of COPD patients. See **Figure 3** for more details.

## 6 Conclusions and future perspectives

IL-5, as a core regulator of eosinophil biological function, has become an important target for the treatment of related diseases. Monoclonal antibodies against IL-5 and its receptor (e.g., mepolizumab, rilizumab) have demonstrated efficacy in clinical trials and in the real world for significantly lowering eosinophil counts, reducing acute exacerbations, and improving lung function and quality of life, particularly in severe eosinophilic asthma, eosinophilic granulomatous polyangiitis, and other diseases. Nevertheless, the complexity of the disease mechanisms and the redundancy of the inflammatory pathways lead to the challenge that single IL-5-targeted therapies still have incomplete responses or limited efficacy in some patients.

The multi-target combination therapy strategy is expected to inhibit the inflammatory network more comprehensively and enhance the therapeutic efficacy by simultaneously intervening IL-5 with other key inflammatory pathways (e.g., IL-4/IL-13, IL-33/ST2, TSLP, etc.). The development of novel therapeutic tools such as bispecific antibodies, small-molecule JAK/STAT inhibitors, and RNA-targeting technology provides new possibilities for breaking through the current therapeutic bottleneck. In addition, individualized treatment strategies based on biomarkers (e.g. blood eosinophil counts, IL-5Rα expression levels, etc.) will help to achieve more precise patient stratification and treatment regimen optimization.

However, a comprehensive clinical appraisal of anti-IL-5 therapies reveals several persistent challenges that warrant cautious optimism. Despite significant therapeutic milestones, a notable subset of patients remains partial responders, potentially due to ‘IL-5-independent’ eosinophilic pathways or the survival of tissue-resident eosinophils that evade systemic depletion. Furthermore, while BEC thresholds provide a framework for precision, the heterogeneous clinical outcomes observed in COPD underscore that eosinophilic inflammation in this context may represent a distinct pathophysiological endotype compared to asthma. Beyond efficacy, long-term safety considerations—specifically the potential impact of chronic eosinophil depletion on parasitic immunity and anti-tumor surveillance—require ongoing pharmacovigilance. Finally, the high economic burden and cost-effectiveness of these biologics remain significant barriers to global access. Future research must therefore focus on refining biomarker-driven selection to maximize the value-to-cost ratio and ensure that these advanced therapies reach the patients who will benefit most.

Future research should further analyse the molecular mechanisms and inflammatory networks of eosinophil-related diseases, promote the clinical validation and translational application of multi-targeted therapeutic regimens, and combine with emerging technologies, such as single-cell multi-omics and artificial intelligence, to promote the continued development of this field towards systematization, precision and individualization, and ultimately to improve the prognosis and quality of life of patients.

## References

[1] Gela A Kasetty G Jovic S Ekoff M Nilsson G Mörgelin M . Eotaxin-3 (CCL26) exerts innate host defense activities that are modulated by mast cell proteases. Allergy. (2015) 70:161–70. doi: 10.1111/all.12542. PMID: 25377782

[2] Hassani M Koenderman L . Immunological and hematological effects of IL-5(Rα)-targeted therapy: An overview. Allergy. (2018) 73:1979–88. doi: 10.1111/all.13451. PMID: 29611207 PMC6220846

[3] LeSuer WE Kienzl M Ochkur SI Schicho R Doyle AD Wright BL . Eosinophils promote effector functions of lung group 2 innate lymphoid cells in allergic airway inflammation in mice. J Allergy Clin Immun. (2023) 152:469–485.e10. doi: 10.1016/j.jaci.2023.03.023. PMID: 37028525 PMC10503660

[4] Zeng LW Feng L Liu R Lin H Shu HB Li S . The membrane-associated ubiquitin ligases MARCH2 and MARCH3 target IL-5 receptor alpha to negatively regulate eosinophilic airway inflammation. Cell Mol Immunol. (2022) 19:1117–29. doi: 10.1038/s41423-022-00907-9. PMID: 35982175 PMC9508171

[5] Taillé C Chanez P Devouassoux G Didier A Pison C Garcia G . Mepolizumab in a population with severe eosinophilic asthma and corticosteroid dependence: results from a French early access programme. Eur Respir J. (2020) 55:1902345. doi: 10.1183/13993003.02345-2019. PMID: 32241829 PMC7315004

[6] Shen ZJ Hu J O'Neal MA Malter JS . Pin1 regulates IL-5 induced eosinophil polarization and migration. Cells-Basel. (2021) 10:211. doi: 10.3390/cells10020211. PMID: 33494375 PMC7910834

[7] Pitlick MM Pongdee T . Combining biologics targeting eosinophils (IL-5/IL-5R), igE, and IL-4/IL-13 in allergic and inflammatory diseases. World Allergy Organ. (2022) 15:100707. doi: 10.1016/j.waojou.2022.100707. PMID: 36267353 PMC9574495

[8] Méndez-García LA Solleiro-Villavicencio H Bueno-Hernández N Cérbulo-Vázquez A Escobedo G Esquivel-Velázquez M . Role of the th2-like immune response in obesity: IL-4 as a metabolic regulator and IL-13 as an effector of muscle energy metabolism. Biomedicines. (2025) 13:2208. doi: 10.3390/biomedicines13092208. PMID: 41007770 PMC12467235

[9] Chikumoto A Oishi K Hamada K Hirano T Kakugawa T Kanesada K . Sequential biotherapy targeting IL-5 and IL-4/13 in patients with eosinophilic asthma with sinusitis and otitis media. Biomolecules. (2022) 12:522. doi: 10.3390/biom12040522. PMID: 35454111 PMC9025540

[10] Nussbaum JC Van Dyken SJ von Moltke J Cheng LE Mohapatra A Molofsky AB . Type 2 innate lymphoid cells control eosinophil homeostasis. Nature. (2013) 502:245–8. doi: 10.1038/nature12526. PMID: 24037376 PMC3795960

[11] Oka A Klingler AI Kidoguchi M Poposki JA Suh LA Bai J . Tezepelumab inhibits highly functional truncated thymic stromal lymphopoietin in chronic rhinosinusitis. J Allergy Clin Immun. (2025) 156:463–467.e2. doi: 10.1016/j.jaci.2025.02.031. PMID: 40057283 PMC12331434

[12] Yousuf A Ibrahim W Greening NJ Brightling CE . T2 biologics for chronic obstructive pulmonary disease. J Aller Cl Imm-Pract. (2019) 7:1405–16. doi: 10.1016/j.jaip.2019.01.036. PMID: 31076058

[13] Gorski SA Lawrence MG Hinkelman A Spano MM Steinke JW Borish L . Expression of IL-5 receptor alpha by murine and human lung neutrophils. PloS One. (2019) 14:e0221113. doi: 10.1371/journal.pone.0221113. PMID: 31415658 PMC6695150

[14] Borish L Teague WG Patrie JT Wavell KW Barros AJ Malpass HC . Further evidence of a type 2 inflammatory signature in chronic obstructive pulmonary disease or emphysema. Ann Allerg Asthma Im. (2023) 130:617–621.e1. doi: 10.1016/j.anai.2023.01.024. PMID: 36736724 PMC10159908

[15] Obeidat M Sadatsafavi M Sin DD . Precision health: treating the individual patient with chronic obstructive pulmonary disease. Med J Aust. (2019) 210:424–8. doi: 10.5694/mja2.50138. PMID: 30977152

[16] Van Eeckhoutte HP Donovan C Kim RY Conlon TM Ansari M Khan H . RIPK1 kinase-dependent inflammation and cell death contribute to the pathogenesis of COPD. Eur Respir J. (2023) 61:2201506. doi: 10.1183/13993003.01506-2022. PMID: 36549711

[17] Vlaykov AN Tacheva TT Vlaykova TI Stoyanov VK . Serum and local IL-4, IL-5, IL-13 and immunoglobulin E in allergic rhinitis. Postep Derm Alergol. (2020) 37:719–24. doi: 10.5114/ada.2020.100483. PMID: 33240012 PMC7675068

[18] Kaganova MM Shilovskiy IP KovChina VI Timotievich ED Rusak TE Nikolskii AA . Suppression of il5 and il13 gene expression by synthetic siRNA molecules reduces nasal hyperreactivity and inflammation in a murine model of allergic rhinitis. Biochemistry-Moscow+. (2025) 90:476–92. doi: 10.1134/S0006297924602946. PMID: 40451198

[19] Sunata K Miyata J Kawashima Y Konno R Ishikawa M Hasegawa Y . Multiomics analysis identified IL-4-induced IL1RL1(high) eosinophils characterized by prominent cysteinyl leukotriene metabolism. J Allergy Clin Immun. (2024) 154:1277–88. doi: 10.1016/j.jaci.2024.07.012. PMID: 39067484

[20] Wang H Fung NH Gamell C Woodman N Chan S Lachapelle P . Signalling via CD131 regulates pulmonary inflammation, alveolar cell death and emphysema in COPD. Erj Open Res. (2025) 11:01020-2024. doi: 10.1183/23120541.01020-2024. PMID: 40551800 PMC12183705

[21] Jiang M Liu H Li Z Wang J Zhang F Cao K . ILC2s induce adaptive th2-type immunity in acute exacerbation of chronic obstructive pulmonary disease. Mediat Inflammation. (2019) 2019:3140183. doi: 10.1155/2019/3140183. PMID: 31320835 PMC6610743

[22] Donovan T Milan SJ Wang R Banchoff E Bradley P Crossingham I . Anti-IL-5 therapies for chronic obstructive pulmonary disease. Cochrane Db Syst Rev. (2020) 12:CD013432. doi: 10.1002/14651858.CD013432.pub2. PMID: 33295032 PMC8106745

[23] Cuevas M Zahnert T . Biologics: A new option in treatment of severe chronic rhinosinusits with nasal polyps. Laryngo Rhino Otol. (2021) 100:134–45. doi: 10.1055/a-1309-6631. PMID: 33525013

[24] Zhang C Wang Y Zhang M Su X Lei T Yu H . Monoclonal antibodies targeting IL-5 or IL-5Rα in eosinophilic chronic obstructive pulmonary disease: A systematic review and meta-analysis. Front Pharmacol. (2021) 12:754268. doi: 10.3389/fphar.2021.754268. PMID: 34795588 PMC8594629

[25] Sun F Zou W Shi H Chen Z Ma D Lin M . Interleukin-33 increases type 2 innate lymphoid cell count and their activation in eosinophilic asthma. Clin Transl Allergy. (2023) 13:e12265. doi: 10.1002/clt2.12265. PMID: 37357549 PMC10234174

[26] Takatsu K . Interleukin-5 and IL-5 receptor in health and diseases. P Jpn Acad B-Phys. (2011) 87:463–85. doi: 10.2183/pjab.87.463. PMID: 21986312 PMC3313690

[27] Burnham ME Esnault S Roti RE Bates ME Bertics PJ Denlinger LC . Cholesterol selectively regulates IL-5 induced mitogen activated protein kinase signaling in human eosinophils. PloS One. (2014) 9:e103122. doi: 10.1371/journal.pone.0103122. PMID: 25121926 PMC4133209

[28] Lyu X Liu J Liu Z Wu Y Zhu P Liu C . Anti-inflammatory effects of reticuline on the JAK2/STAT3/SOCS3 and p38 MAPK/NF-κB signaling pathway in a mouse model of obesity-associated asthma. Clin Respir J. (2024) 18:e13729. doi: 10.1111/crj.13729. PMID: 38286741 PMC10799233

[29] Yu H Huang X Xie C Song J Zhou Y Shi H . Transcriptomics reveals apigenin alleviates airway inflammation and epithelial cell apoptosis in allergic asthma via MAPK pathway. Phytother Res. (2023) 37:4002–17. doi: 10.1002/ptr.7859. PMID: 37128812

[30] Li Y Yu HY Zhao KC Ding XH Huang Y Hu SP . Effects of medication use on small airway function and airway inflammation in patients with clinically controlled asthma. Curr Med Sci. (2021) 41:722–8. doi: 10.1007/s11596-021-2403-5. PMID: 34403097

[31] Sun N Ogulur I Mitamura Y Yazici D Pat Y Bu X . The epithelial barrier theory and its associated diseases. Allergy. (2024) 79:3192–237. doi: 10.1111/all.16318. PMID: 39370939 PMC11657050

[32] David B Bafadhel M Koenderman L De Soyza A . Eosinophilic inflammation in COPD: from an inflammatory marker to a treatable trait. Thorax. (2021) 76:188–95. doi: 10.1136/thoraxjnl-2020-215167. PMID: 33122447 PMC7815887

[33] Yun JH Lamb A Chase R Singh D Parker MM Saferali A . Blood eosinophil count thresholds and exacerbations in patients with chronic obstructive pulmonary disease. J Allergy Clin Immun. (2018) 141:2037–2047.e10. doi: 10.1016/j.jaci.2018.04.010. PMID: 29709670 PMC5994197

[34] Cabrera LC Sánchez SA Lemes CA Cazorla RS Breña AJ González DE . Eosinophil subtypes in adults with asthma and adults with chronic obstructive pulmonary disease. Am J Resp Crit Care. (2023) 208:155–62. doi: 10.1164/rccm.202301-0149OC. PMID: 37071848

[35] Fieldes M Bourguignon C Assou S Nasri A Fort A Vachier I . Targeted therapy in eosinophilic chronic obstructive pulmonary disease. Erj Open Res. (2021) 7:00437-2020. doi: 10.1183/23120541.00437-2020. PMID: 33855061 PMC8039900

[36] Uppal V Kreiger P Kutsch E . Eosinophilic gastroenteritis and colitis: a comprehensive review. Clin Rev Allerg Immu. (2016) 50:175–88. doi: 10.1007/s12016-015-8489-4. PMID: 26054822

[37] Prussin C Lee J Foster B . Eosinophilic gastrointestinal disease and peanut allergy are alternatively associated with IL-5+ and IL-5(-) T(H)2 responses. J Allergy Clin Immun. (2009) 124:1326–1332.e6. doi: 10.1016/j.jaci.2009.09.048. PMID: 20004787 PMC2994258

[38] Kim YJ Prussin C Martin B Law MA Haverty TP Nutman TB . Rebound eosinophilia after treatment of hypereosinophilic syndrome and eosinophilic gastroenteritis with monoclonal anti-IL-5 antibody SCH55700. J Allergy Clin Immun. (2004) 114:1449–55. doi: 10.1016/j.jaci.2004.08.027. PMID: 15577851

[39] Bischoff SC . Food allergy and eosinophilic gastroenteritis and colitis. Curr Opin Allergy Cl. (2010) 10:238–45. doi: 10.1097/ACI.0b013e32833982c3. PMID: 20431371

[40] Song DJ Shim MH Lee N Yoo Y Choung JT . CCR3 monoclonal antibody inhibits eosinophilic inflammation and mucosal injury in a mouse model of eosinophilic gastroenteritis. Allergy Asthma Immun. (2017) 9:360–7. doi: 10.4168/aair.2017.9.4.360. PMID: 28497923 PMC5446951

[41] Kotepui M Duangchan T Mahittikorn A Mekhora C Anabire NG Kotepui KU . Interleukin-5 levels in relation to malaria severity: a systematic review. Malaria J. (2023) 22:226. doi: 10.1186/s12936-023-04659-3. PMID: 37537570 PMC10401852

[42] Singh D Fuhr R Bird NP Mole S Hardes K Man YL . A Phase 1 study of the long-acting anti-IL-5 monoclonal antibody GSK3511294 in patients with asthma. Brit J Clin Pharmaco. (2022) 88:702–12. doi: 10.1111/bcp.15002. PMID: 34292606 PMC9290054

[43] Carpagnano GE Portacci A Nolasco S Detoraki A Vatrella A Calabrese C . Features of severe asthma response to anti-IL5/IL5r therapies: identikit of clinical remission. Front Immunol. (2024) 15:1343362. doi: 10.3389/fimmu.2024.1343362. PMID: 38327518 PMC10848329

[44] Harish A Schwartz SA . Targeted anti-IL-5 therapies and future therapeutics for hypereosinophilic syndrome and rare eosinophilic conditions. Clin Rev Allerg Immu. (2020) 59:231–47. doi: 10.1007/s12016-019-08775-4. PMID: 31919743

[45] Van Hulst G Jorssen J Jacobs N Henket M Louis R Schleich F . Anti-IL5 mepolizumab minimally influences residual blood eosinophils in severe asthma. Eur Respir J. (2022) 59:2100935. doi: 10.1183/13993003.00935-2021. PMID: 34475229

[46] Roufosse F Kahn JE Rothenberg ME Wardlaw AJ Klion AD Kirby SY . Efficacy and safety of mepolizumab in hypereosinophilic syndrome: A phase III, randomized, placebo-controlled trial. J Allergy Clin Immun. (2020) 146:1397–405. doi: 10.1016/j.jaci.2020.08.037. PMID: 32956756 PMC9579892

[47] Chen XQ Jia XY Wu JJ Huang M Sun W Ji N . Efficacy and safety of omalizumab in patients with refractory allergic asthma: a meta-analysis. Zhonghua Yi Xue Za Zhi. (2022) 102:2201–9. doi: 10.3760/cma.j.cn112137-20211109-02480. PMID: 35872585

[48] Pavord ID Bel EH Bourdin A Chan R Han JK Keene ON . From DREAM to REALITI-A and beyond: Mepolizumab for the treatment of eosinophil-driven diseases. Allergy. (2022) 77:778–97. doi: 10.1111/all.15056. PMID: 34402066 PMC9293125

[49] FitzGerald JM Bleecker ER Menzies-Gow A Zangrilli JG Hirsch I Metcalfe P . Predictors of enhanced response with benralizumab for patients with severe asthma: pooled analysis of the SIROCCO and CALIMA studies. Lancet Resp Med. (2018) 6:51–64. doi: 10.1016/S2213-2600(17)30344-2. PMID: 28919200

[50] Alvarado-Vazquez PA Mendez-Enriquez E Salomonsson M Kopac P Koren A Bidovec-Stojkovic U . Targeting of the IL-5 pathway in severe asthma reduces mast cell progenitors. J Allergy Clin Immun. (2025) 155:1310–20. doi: 10.1016/j.jaci.2024.10.025. PMID: 39521285

[51] Menzella F Marchi M Caminati M Romagnoli M Micheletto C Bonato M . Long-term eosinophil depletion: A real-world perspective on the safety and durability of benralizumab treatment in severe eosinophilic asthma. J Clin Med. (2024) 14:191. doi: 10.3390/jcm14010191. PMID: 39797273 PMC11722057

[52] Pelaia G Jackson DJ Nair P Emmanuel B Tran TN Menzies-Gow A . XALOC-1: Clinical remission over 2 years with benralizumab in severe eosinophilic asthma. Chest. (2025) 168:19–32. doi: 10.1016/j.chest.2025.04.011. PMID: 40258512

[53] Louis R Lommatzsch M Jackson DJ Menzies-Gow A Shavit A Cohen D . Advancing remission in severe asthma with benralizumab: Latest findings, current perspectives and future direction. Clin Exp Allergy. (2025) 55:521–31. doi: 10.1111/cea.70083. PMID: 40444568 PMC12221862

[54] Hirose M Kuwabara K Kondo R Horiguchi T . A prospective study exploring the predictors of response to benralizumab in patients with refractory bronchial asthma. Fujita Med J. (2022) 8:13–6. doi: 10.20407/fmj.2020-024. PMID: 35233342 PMC8874918

[55] Bleecker ER FitzGerald JM Chanez P Papi A Weinstein SF Barker P . Efficacy and safety of benralizumab for patients with severe asthma uncontrolled with high-dosage inhaled corticosteroids and long-acting β(2)-agonists (SIROCCO): a randomised, multicentre, placebo-controlled phase 3 trial. Lancet. (2016) 388:2115–27. doi: 10.1016/S0140-6736(16)31324-1. PMID: 27609408

[56] Pini L Bagnasco D Beghè B Braido F Cameli P Caminati M . Unlocking the long-term effectiveness of benralizumab in severe eosinophilic asthma: A three-year real-life study. J Clin Med. (2024) 13:3013. doi: 10.3390/jcm13103013. PMID: 38792553 PMC11122375

[57] Kavanagh JE Hearn AP Dhariwal J D'Ancona G Douiri A Roxas C . Real-world effectiveness of benralizumab in severe eosinophilic asthma. Chest. (2021) 159:496–506. doi: 10.1016/j.chest.2020.08.2083. PMID: 32882249

[58] Ramakrishnan S Camp JR Vijayakumar B Hardinge FM Downs ML Russell R . The use of benralizumab in the treatment of near-fatal asthma: A new approach. Am J Resp Crit Care. (2020) 201:1441–3. doi: 10.1164/rccm.202001-0093LE. PMID: 32023077 PMC7258629

[59] Lee H Neri P Bahlis NJ . BCMA- or GPRC5D-targeting bispecific antibodies in multiple myeloma: Efficacy, safety, and resistance mechanisms. Blood. (2024) 143:1211–7. doi: 10.1182/blood.2023022499. PMID: 38194680

[60] Neri P Leblay N Lee H Gulla A Bahlis NJ Anderson KC . Just scratching the surface: Novel treatment approaches for multiple myeloma targeting cell membrane proteins. Nat Rev Clin Oncol. (2024) 21:590–609. doi: 10.1038/s41571-024-00913-y. PMID: 38961233

[61] Isozaki T Homma T Sagara H Kasama T . Role of cytokines in EGPA and the possibility of treatment with an anti-IL-5 antibody. J Clin Med. (2020) 9:3890. doi: 10.3390/jcm9123890. PMID: 33265990 PMC7760889

[62] Li S Wang S Fordjour E Liang Y Wang X Ye Y . Development and characterization of anti-IL-5 monoclonal antibody Fab fragment for blocking IL-5/IL-5Rα binding. Int Immunopharmacol. (2023) 124:111032. doi: 10.1016/j.intimp.2023.111032. PMID: 37832239

[63] Wang Y Cao Z Zhao H Gu Z . Nonylphenol exacerbates ovalbumin-induced allergic rhinitis via the TSLP-TSLPR/IL-7R pathway and JAK1/2-STAT3 signaling in a mouse model. Ecotox Environ Safe. (2022) 243:114005. doi: 10.1016/j.ecoenv.2022.114005. PMID: 36029577

[64] Zhu Y Chen L Huang Z Alkan S Bunting KD Wen R . Cutting edge: IL-5 primes Th2 cytokine-producing capacity in eosinophils through a STAT5-dependent mechanism. J Immunol. (2004) 173:2918–22. doi: 10.4049/jimmunol.173.5.2918. PMID: 15322148

[65] Ye W Fan C Fu K Wang X Lin J Nian S . The SAR and action mechanisms of autophagy inhibitors that eliminate drug resistance. Eur J Med Chem. (2022) 244:114846. doi: 10.1016/j.ejmech.2022.114846. PMID: 36283182

[66] Nakai Y Ohashi Y Kakinoki Y Tanaka A Washio Y Nasako Y . Allergen-induced mRNA expression of IL-5, but not of IL-4 and IFN-gamma, in peripheral blood mononuclear cells is a key feature of clinical manifestation of seasonal allergic rhinitis. Arch Otolaryngol Head Neck Surg. (2000) 126:992–6. doi: 10.1001/archotol.126.8.992. PMID: 10922233

[67] Shen C Wu N Chen X Peng J Feng M Wang J . Interleukin-5 alleviates cardiac remodelling via the STAT3 pathway in angiotensin II-infused mice. J Cell Mol Med. (2024) 28:e18493. doi: 10.1111/jcmm.18493. PMID: 38963241 PMC11223166

[68] Luo J Chen W Liu W Jiang S Ye Y Shrimanker R . IL-5 antagonism reverses priming and activation of eosinophils in severe eosinophilic asthma. Mucosal Immunol. (2024) 17:524–36. doi: 10.1016/j.mucimm.2024.03.005. PMID: 38493955 PMC11649845

[69] Milne ME Kimball J Tarrant TK Al-Rohil RN Leverenz DL . The role of T helper type 2 (Th2) cytokines in the pathogenesis of eosinophilic granulomatosis with polyangiitis (eGPA): An illustrative case and discussion. Curr Allergy Asthm R. (2022) 22:141–50. doi: 10.1007/s11882-022-01039-w. PMID: 36103081 PMC9471022

[70] D'Arcy Q Gharaee-Kermani M Zhilin-Roth A Macoska JA . The IL-4/IL-13 signaling axis promotes prostatic fibrosis. PloS One. (2022) 17:e0275064. doi: 10.1371/journal.pone.0275064. PMID: 36201508 PMC9536598

[71] Ho T Zhai T Chen Y Dai S Zhou S Zhang Z . BBT002: A novel tetravalent bispecific antibody targeting IL4Ra and IL5 for enhanced asthma therapy. Am J Resp Crit Care. (2025) 211:A1302. doi: 10.1164/ajrccm.2025.211.abstracts.a1302. PMID: 39867992

[72] Xiaochun L Cuizhen L Xiujuan L Xiangyu X Wei KE Zhenwen Q . Effect of improved Yupingfeng powder prescription on interleukin-33/suppression of tumorigenicity 2 pathway in mice with ovalbumins-induced allergic rhinitis. J Tradit Chin Med. (2025) 45:1215–27. doi: 10.19852/j.cnki.jtcm.2025.06.004. PMID: 41376215 PMC12711636

[73] Okragly AJ Corwin KB Elia M He D Schroeder O Zhang Q . Generation and characterization of torudokimab (LY3375880): A monoclonal antibody that neutralizes interleukin-33. J Inflammation Res. (2021) 14:3823–35. doi: 10.2147/JIR.S320287. PMID: 34408465 PMC8364917

[74] Boberg E Johansson K Malmhäll C Calvén J Weidner J Rådinger M . Interplay between the IL-33/ST2 axis and bone marrow ILC2s in protease allergen-induced IL-5-dependent eosinophilia. Front Immunol. (2020) 11:1058. doi: 10.3389/fimmu.2020.01058. PMID: 32582171 PMC7280539

[75] Toki S Goleniewska K Zhang J Zhou W Newcomb DC Zhou B . TSLP and IL-33 reciprocally promote each other's lung protein expression and ILC2 receptor expression to enhance innate type-2 airway inflammation. Allergy. (2020) 75:1606–17. doi: 10.1111/all.14196. PMID: 31975538 PMC7354889

[76] Malik B Bartlett NW Upham JW Nichol KS Harrington J Wark P . Severe asthma ILC2s demonstrate enhanced proliferation that is modified by biologics. Respirology. (2023) 28:758–66. doi: 10.1111/resp.14506. PMID: 37114915 PMC10946917

[77] Sezgin ME Çolak M Çağlayan Ö Yumrukuz ŞM Aktepe SE Çoban H . Efficacy of mepolizumab and omalizumab combination therapy in uncontrolled asthma. J Asthma. (2024) 61:173–5. doi: 10.1080/02770903.2023.2244590. PMID: 37530447

[78] Matsuda M Shimora H Nakayama Y Matsumura M Kitao A Ariyoshi Y . Essential roles of mechanistic target of rapamycin in the induction of steroid resistance in group 2 innate lymphoid cells and severe asthma. J Pharmacol Exp Ther. (2025) 392:103744. doi: 10.1016/j.jpet.2025.103744. PMID: 41172625

[79] Bendien SA Kroes JA van Hal L Braunstahl GJ Broeders M Oud K . Real-world effectiveness of IL-5/5Ra targeted biologics in severe eosinophilic asthma with comorbid bronchiectasis. J Aller Cl Imm-Pract. (2023) 11:2724–2731.e2. doi: 10.1016/j.jaip.2023.05.041. PMID: 37295671

[80] Meng G Cui H Feng C Guo C Song L Duan Z . Oral administration of Limosilactobacillus reuteri VHProbi® M07 alleviates ovalbumin-induced allergic asthma in mice. PloS One. (2025) 20:e0317587. doi: 10.1371/journal.pone.0317587. PMID: 39820222 PMC11737801

[81] Reyes-Pavón D Cervantes-García D Bermúdez-Humarán LG Córdova-Dávalos LE Quintanar-Stephano A Jiménez M . Protective effect of glycomacropeptide on food allergy with gastrointestinal manifestations in a rat model through down-regulation of type 2 immune response. Nutrients. (2020) 12:2942. doi: 10.3390/nu12102942. PMID: 32992996 PMC7601722

[82] El-Alali EA Abukhiran IM Alhmoud TZ . Successful use of montelukast in eosinophilic gastroenteritis: A case report and a literature review. BMC Gastroenterol. (2021) 21:279. doi: 10.1186/s12876-021-01854-x. PMID: 34238222 PMC8265096

[83] Kanamori A Tanaka F Ominami M Nadatani Y Fukunaga S Otani K . Psychological stress exacerbates inflammation of the ileum via the corticotropin-releasing hormone-mast cell axis in a mouse model of eosinophilic enteritis. Int J Mol Sci. (2022) 23:8538. doi: 10.3390/ijms23158538. PMID: 35955675 PMC9369025

[84] Sunata K Miyata J Kawashima Y Konno R Ishikawa M Hasegawa Y . Inflammatory profile of eosinophils in asthma-COPD overlap and eosinophilic COPD: A multi-omics study. Front Immunol. (2024) 15:1445769. doi: 10.3389/fimmu.2024.1445769. PMID: 39439801 PMC11493663

[85] Varricchi G Poto R . Towards precision medicine in COPD: Targeting type 2 cytokines and alarmins. Eur J Intern Med. (2024) 125:28–31. doi: 10.1016/j.ejim.2024.05.011. PMID: 38762432

[86] Alfaro A Vargas H Díaz JM Pinto JA Cambero D Cuellar EH . Anti-IL-5 and anti-IL-5 receptor therapy significantly improves quality of life and FEV1 values in patients with severe asthma. Allergy Asthma Cl Im. (2025) 21:34. doi: 10.1186/s13223-025-00979-y. PMID: 40804423 PMC12351985

[87] Broide D . Targeting eosinophils in asthmatic inflammation: Benefits and drawbacks. J Inflammation Res. (2025) 18:12421–45. doi: 10.2147/JIR.S521238. PMID: 40951444 PMC12433234

[88] Kang HS Kim SK Kim YH Kim JW Lee SH Yoon HK . The association between eosinophilic exacerbation and eosinophilic levels in stable COPD. BMC Pulm Med. (2021) 21:74. doi: 10.1186/s12890-021-01443-4. PMID: 33653314 PMC7923497

[89] Pavord ID Chapman KR Bafadhel M Sciurba FC Bradford ES Schweiker HS . Mepolizumab for eosinophil-associated COPD: analysis of METREX and METREO. Int J Chronic Obstr. (2021) 16:1755–70. doi: 10.2147/COPD.S294333. PMID: 34163157 PMC8215850

